# Effects of home disease management strategies based on the dyadic illness management theory on elderly patients with chronic heart failure and informal caregivers’ physical and psychological outcomes: a randomized controlled trial

**DOI:** 10.3389/fmed.2025.1679743

**Published:** 2025-11-18

**Authors:** Taihua Zhou, Yaoyao Hu, Lingyun Zhu, Ping Zhu, Xingxing Ji, Jia Wang, Hui Shang, Chence Zhao

**Affiliations:** 1 Affiliated Hospital of Jiangnan University, Wuxi, China; 2 Department of Nursing, Beijing Health Vocational College, Beijing, China

**Keywords:** chronic heart failure, disease management, dyadic health, informal caregivers, quality of life, readmission

## Abstract

**Objective:**

The purpose of our study was to consider elderly patients with chronic heart failure (CHF) and informal caregivers as a unit of intervention and to explore the effectiveness of a program developed based on dyadic illness management theory in improving their physical and psychological health.

**Methods:**

In this single-center randomized controlled trial, 80 dyads of elderly CHF patients and their informal caregivers were randomly assigned (1:1) to an intervention group receiving a 3-month home-based disease management program or a control group receiving usual care. Data on primary outcome (patient QoL) and secondary outcomes (e.g., patient self-management, readmission, depression, and caregiver burden) were collected at baseline (T0), immediately post-intervention (T1), and 3 months post-intervention (T2).

**Results:**

At both T1 and T2, patients in the intervention group showed significantly better outcomes than controls in QoL, readmission rates, self-care behaviors, and depression incidence (all *p* < 0.05). Caregiver burden was consistently lower in the intervention group at both timepoints (all *p* < 0.05). QoL showed temporal improvements in the intervention group, with sustained enhancement at both T1 and T2 (compared to baseline) and further progression from T1 to T2. In contrast, the control group demonstrated only transient improvement at T1 (compared to baseline), followed by decline at T2. After adjusting for baseline factors, the intervention demonstrated a significant and independent effect on improving patient QoL. This effect was not only sustained but substantially strengthened from T1 (*B* = −6.855) to T2 (*B* = -25.00, both *p* < 0.001), indicating a cumulative benefit over time. The model for T2 accounted for the majority of the outcome variance (adjusted *R*^2^ = 0.828).

**Conclusion:**

The home-based, dyadically-focused disease management program significantly improved both physical and psychological health outcomes for elderly CHF patients and their caregivers. And substantially increasing improvements in patients’ QoL.

## 1 Introduction

Demographic aging and rising cardiovascular risks are contributing to a rising prevalence of chronic heart failure (CHF) among the elderly, where it is a leading cause of hospitalization and a critical public health challenge (1–4). As a chronic condition, CHF necessitates effective long-term management after hospitalization. International guidelines emphasize that patients with chronic heart failure require management during home care to control their condition, improve symptoms, enhance quality of life (QoL), and reduce recurrence rates and mortality (5–7). This has led to an increasing emphasis on home-based disease management, which refers to the comprehensive suite of care activities, including both patient self-management and caregiver support, conducted within the home environment (8, 9). Evidences confirms that optimized home management significantly enhances patient outcomes and may lower healthcare costs (10, 11). However, factors such as advanced age, comorbidities, and low comprehension often make treatment adherence burdensome for elderly CHF patients, leading to a generally suboptimal level of home-based disease management in China (11–14). Consequently, improving disease management capabilities in home care settings has emerged as a critical challenge requiring urgent attention.

The European Society of Cardiology highlights patient and family involvement in home-based disease management as vital for successful treatment (6), with head and neck cancer research confirming that enhanced caregiver education improves outcomes (15). Despite these benefits, family caregivers endure significant physical, psychological, and social strain (16). In China, CHF caregivers face heightened burdens due to inadequate knowledge, skills, and social support (17, 18), undermining care efficacy and emphasizing the urgent need for comprehensive training and professional support systems. In the home-based management of elderly CHF patients, informal caregivers (ICs) serve a critical role yet encounter substantial caregiving challenges. Addressing these challenges requires a dual focus: (1) meeting caregivers’ daily needs, and (2) developing targeted interventions to enhance patient and caregiver support.

Dyadic interventions, in which both the patient and family caregiver are targeted, are likely to result in better outcomes for both and are more cost-effective than interventions with a single objective (19). The theory of dyadic illness management reconceptualizes chronic disease management as an interdependent process between the patient and caregiver. This paradigm shifts the focus from the individual to the dyad, emphasizing that successful management is a bidirectional phenomenon, reliant on their collaborative assessment and health-promoting behaviors to achieve optimal mutual outcomes (20). Its core components include: Dyadic appraisal (joint evaluation of symptoms, care goals, and decision-making); Dyadic management behaviors (collaborative actions such as shared decision-making, daily health management, and emotional support); Dyadic health (an interdependent relationship encompassing both parties’ physical/mental well-being and QoL). These elements interact cyclically: appraisal influences behaviors, behaviors determine health outcomes, and health status reciprocally modifies appraisal and management. From a practical standpoint, dyadic interventions targeting both patients and family caregivers prove more cost-effective and yield better outcomes for both parties than patient-only approaches (21).

This approach, which addresses the dyad as an interdependent unit, is supported internationally by evidence of improved communication, emotional regulation, and disease management skills (22). Exploratory studies in the Chinese context further confirm benefits in discharge readiness and psychosocial outcomes for dyads facing lung cancer or heart failure (23, 24). Therefore, nursing interventions designed to target this dyadic cycle are crucial for achieving sustainable health outcomes. Despite this evidence, the application of such integrated dyadic models in CHF care for older adults in China remains limited, highlighting a significant area for development.

Nevertheless, in the context of heart failure management in China, research remains largely focused on patients or caregivers individually, with limited attention to the patient–caregiver dyad as an integrated unit, particularly among older adults. Moreover, existing self-management interventions for CHF patients predominantly consist of in-hospital health education, lacking continuity into home settings. They also fail to address the progressive decline in treatment adherence and self-management after discharge, compounded by the common absence of sustained health education beyond hospitalization (25). Therefore, this study implemented a home-based disease management intervention program grounded in dyadic illness management theory for elderly patients with CHF and their informal caregivers, aiming to examine its effects on the physical and mental health outcomes of both patient-caregiver dyads. We hypothesized that this dyadic approach would significantly improve physical and mental health outcomes for CHF patients and their informal caregivers.

## 2 Methods

### 2.1 Aim

The primary objectives of this study are to evaluate whether home-based disease management can significantly improve QoL for CHF patients, while secondary objectives aim to examine its effects on enhancing patients’ self-management behaviors, reducing readmission rates, alleviating depression, as well as reducing caregiving burden among informal caregivers.

### 2.2 Study design

**Figure 1:**
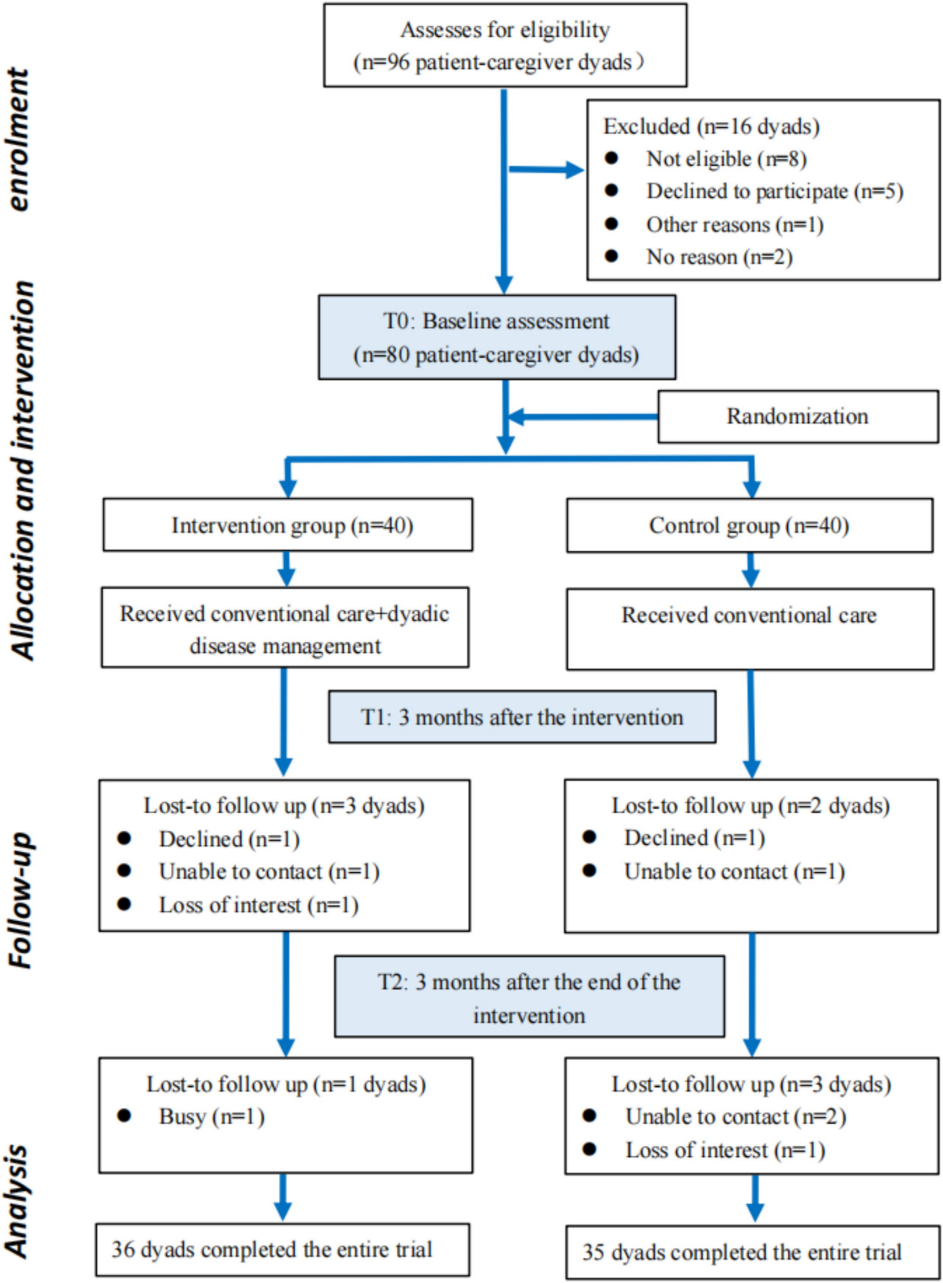
Flow chart of the study. No reason indicates cases where participants did not respond to invitations (e.g., unreturned calls, no replies to messages) and no explicit reason for non-participation was obtained.

This was a two-arm parallel randomized controlled trial (26), which was conducted in the Department of Cardiology, Jiangnan University Hospital from May 2023 to December 2024. A total of 80 dyadic pairs of elderly patients with CHF and their caregivers were randomly divided into an intervention group (*n* = 40 dyads) and a control group (*n* = 40 dyads). Given the inherent characteristics of the intervention, participant blinding was impossible. Nevertheless, blinding was strictly enforced for all outcome assessment and data handling processes to minimize bias. To this end: Outcome assessors were personnel independent of the intervention team and were not informed of the group assignments. Data collection forms used for outcome measures did not identify the study group. The principal statistician performed the final analysis on a dataset with concealed group codes. The control group was given routine nursing care including in-hospital health promotion and post-discharge follow-up, while the intervention group received routine nursing care along with a home-based disease management intervention strategy based on the dyadic illness management theory for a period of 3 months. The flow chart is shown in Figure 1.

### 2.3 Participants

The inclusion and exclusion criteria for subjects are shown in Table 1.

**Table 1:** Inclusion and exclusion criteria for subjects.

Subjects	Criteria for inclusion	Criteria for exclusion
Patients	Those were confirmed diagnosis of CHF with non-end-stage disease (NYHA Class I-III) Aged 60 years or older Capable of providing written informed consent (as demonstrated by signed consent documentation) Intact communicative function and adequate health literacy, defined as the ability to describe symptoms, understand investigator’s questions, and comprehend study procedures and questionnaires Functional proficiency in smartphone operation (e.g., able to install and use mobile applications, and complete digital questionnaires)	Patients with comorbid life-threatening conditions (e.g., active malignancy, end-stage liver/renal disease, hematologic malignancies, or complete loss of independence) Were currently enrolled in other clinical trials Previous formal training in CHF self-management principles, to avoid prior knowledge bias Scheduled relocation from Wuxi during the study, compromising follow-up
Informal caregivers	Primary family caregiver of the enrolled CHF patient Aged 18 years or older Providing ≥8 h of daily care for ≥5 days weekly Competent in smartphone operation Capacity to provide written informed consent (documented through signed consent forms) Intact communicative function with adequate health literacy for protocol comprehension	Those were currently enrolled in other clinical trials Had received training in CHF management Anticipated relocation from the study area during trial period

CHF, Chronic heart failure; NYHA, New York Heart Association.

### 2.4 Randomization

A computer-generated randomization sequence was created using SPSS 27.0 by independent personnel not involved in the study. Eligible patient-caregiver dyads who met the inclusion criteria and provided informed consent were sequentially assigned to their respective groups through sealed, opaque envelopes containing pre-generated allocation codes. Before the intervention was implemented, the control group and intervention group were assigned to different wards.

### 2.5 Intervention

Participants in the control group received routine nursing care, whereas those in the intervention group were administered home disease management programs.

#### 2.5.1 Control group

**Figure 2:**
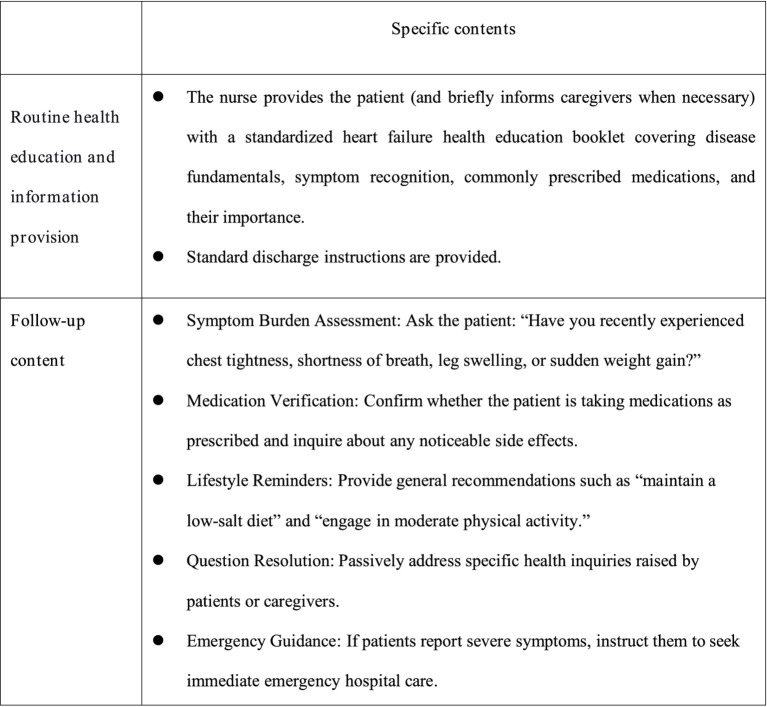
Intervention measures for the control group.

The control group received routine nursing care, discharge instructions, and follow-up care. In-hospital health education: Health education was conducted by department doctors, using a combination of power point presentations and video playback to transmit CHF-related knowledge and disease management points to patients and their informal caregivers. At the end of the process of education, they were given the corresponding disease self-management manual. Post-discharge follow-up: Conducted by two trained research nurses through multiple modalities including structured telephone interviews, WeChat-based communication (via the instant messaging platform), and scheduled home visits. Patients were followed up once a week for the first month after discharge, and then once a month thereafter. Each follow-up visit lasted approximately 5–10 min. During follow-up visits, nurses responded to patients’ specific inquiries, conducted routine condition assessments (such as checking for worsening symptoms like edema or dyspnea, and measuring vital signs during home visits), reminded patients to take their medications, and confirmed their next outpatient appointment date. Details are shown in Figure 2.

#### 2.5.2 Intervention group

**Figure 3:**
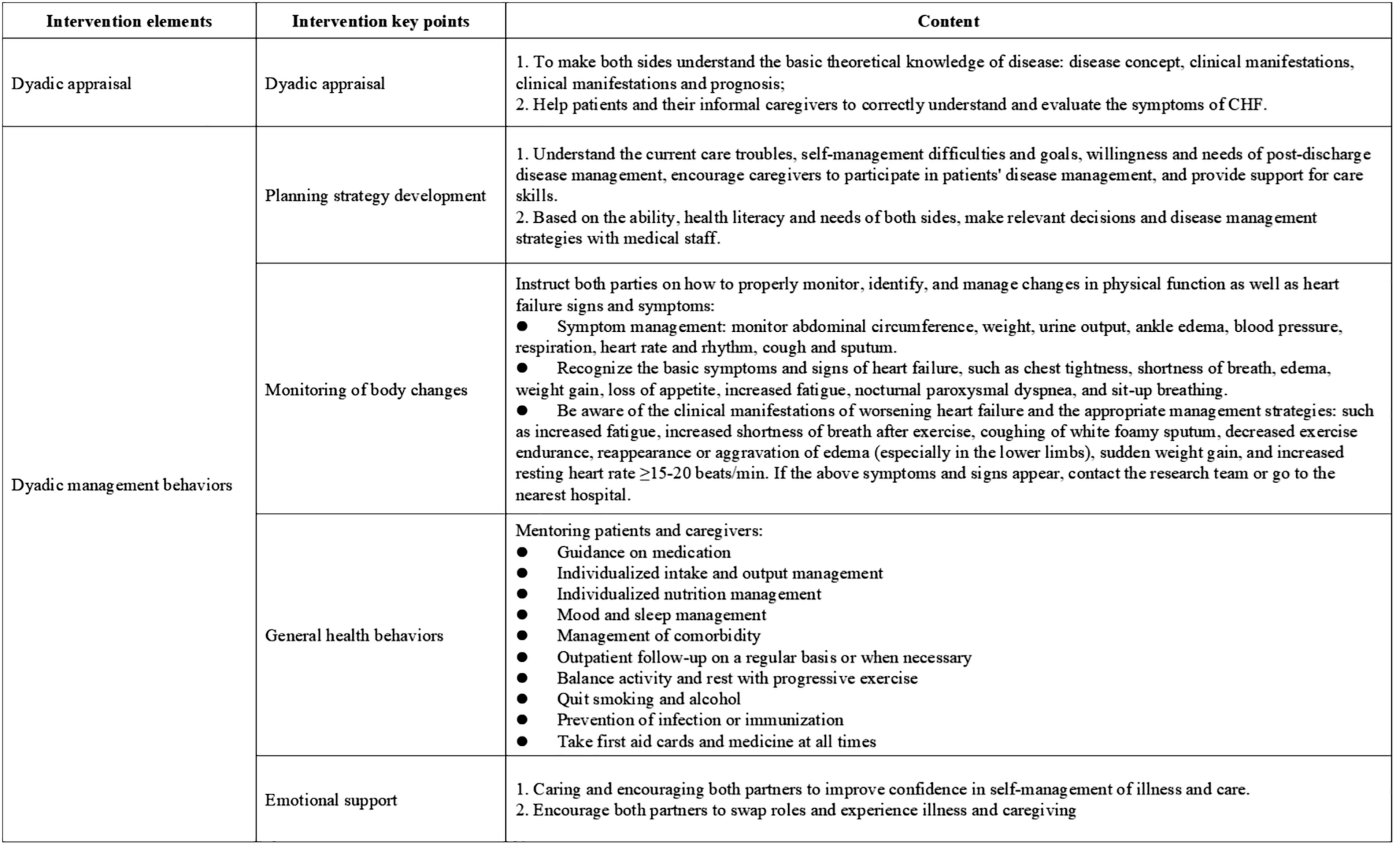
Key implementation considerations for home-based disease management.

Guided by an integrated theoretical framework incorporating Dyadic Illness Management theory (20), Timing It Right theory (27), the Chronic Illness Trajectory Model (28), and relevant clinical practice guideline, this study developed a home-based disease management strategy for elderly CHF patients and their caregivers. The intervention was systematically designed to align with CHF treatment and rehabilitation phases, with detailed components presented in Figure 3. The intervention protocol comprised two sequential phases spanning 12 weeks (7 total sessions). Phase I (Weeks 0–2) delivered 2 face-to-face sessions (60 min each) during hospitalization, focusing on acute care transition and dyadic skill-building. Phase II (Weeks 2–12) implemented 5 biweekly telehealth sessions via Tencent Meeting platform (60 ± 5 min each), emphasizing chronic disease self-management.

### 2.6 Measurements and data collection

**Figure 4:**
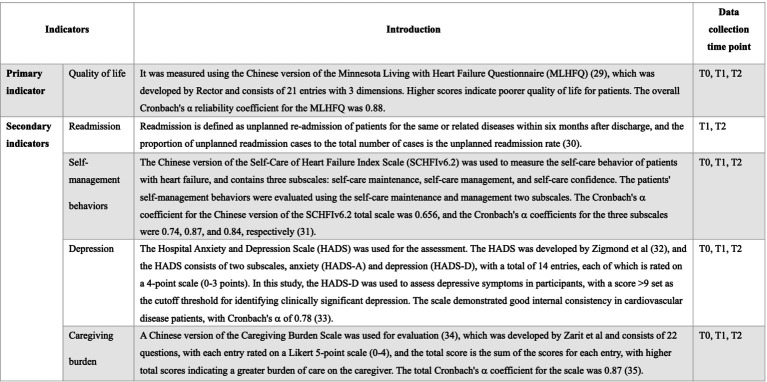
Research tools and time points for data collection. T0: Baseline (pre-intervention); T1: At the end of the intervention, T2: 3 months after the end of the intervention.

The primary outcome indicator of this study was the patients’ QoL. Secondary outcome measures included patients’ readmission rates, self-management behaviors, depression, and caregivers’ burden of care. The assessment instruments and the time taken to collect information are shown in Figure 4.

### 2.7 Adherence

Participant adherence is a critical factor in ensuring the validity of research data, the smooth implementation of interventions, and the reliability of conclusions. We clearly informed participants of the importance of compliance for improving personal health and the scientific validity of the study, thereby reducing the dropout rate. In addition, during the research process, we simplified the data collection process by scheduling follow-up visits at times convenient for participants and using electronic records to replace cumbersome paper forms. We also provide periodic reminders via phone calls and text messages to promptly address participants’ questions. We also offer appropriate positive incentives (such as health consultation services or gifts) to individuals who consistently participate to enhance their continued engagement.

### 2.8 Statistical analyses

For calculating the sample size, the sample size formula for comparing the means of two samples was used. With the specific formula (36) given by: n₁ = n₂ = 2[(Z_*α*/2_ + Z*_β_*)^2^*σ*^2^]/δ^2^. Z_α_ and Z_β_ represent the critical values from the standard normal distribution corresponding to *α* = 0.05 (two-tailed test) and *β* = 0.20, respectively. Using QoL in CHF patients as the primary outcome, based on previous literature (24), the standard deviation (σ) of the outcome measure was set at 5.01, while the expected between-group mean difference (*δ*) was estimated as 8.15, with a significance level of α = 0.05 and statistical power of 1-β = 0.8, the calculation showed that at least 33 patients per group were required. In order to ensure sufficient statistical power, the study increased the sample size to 40 participants per group (a total of 80 patients) and included 40 matched caregivers per group (a total of 80 caregivers), accounting for a possible 20% attrition rate.

SPSS 27.0 statistical software was used for data analysis. Continuous variables are presented as mean ± standard deviation (SD) or median with interquartile range (IQR), depending on data distribution. Analysis of differences between or within groups: independent samples t-test, paired t-test, repeated measures ANOVA, Mann–Whitney U-test, depending on the distribution of data. Categorical variables are presented as frequencies and percentages. Application of chi-square test, Fisher’s exact test and Wilcoxon rank sum test for comparison of categorical variables. To delineate the independent effect of the home-based disease management intervention on the QoL of elderly patients with CHF while controlling for potential confounders, this study employed multiple linear regression analyses. The dependent variables were QoL scores at T1 and T2. The core independent variable was the group assignment to the intervention (0 = No, 1 = Yes). Confounding variables adjusted for included demographic characteristics, disease-related features, and care-related factors. Statistical significance was set at two-sided *p* < 0.05.

## 3 Results

A total of 96 CHF patient-caregiver dyads were screened for this study. After screening, 8 dyads were excluded for failing to meet the inclusion criteria, another 5 declined to participate, and 1 was excluded for other reasons. Eventually, 80 eligible dyads were enrolled and randomly assigned to either the intervention group (n = 40 dyads) or the control group (n = 40 dyads). All enrolled dyads completed the baseline assessment. During subsequent follow-ups, 75 dyads were retained at time point T1 and 71 at time point T2. The flow of participants throughout the study process was detailed in Figure 1.

### 3.1 Comparison of baseline data between the two groups

The data of 36 dyads of participants in the experimental group and 35 participants in the control group were finally included in this study for statistical analysis. As shown in Tables 2, 3, there were no statistically significant differences in baseline data between the two groups.

**Table 2:** Baseline data comparison between the two CHF patient groups.

Variables	CHF patients		Test value	*p* value
	Intervention group (*n* = 36)	Control group (*n* = 35)		
Age (years)	69.86 ± 8.58	71.89 ± 8.12	1.021^a^	0.311
Body mass index (kg/m^2^)	25.56 ± 2.61	24.99 ± 3.20	0.821^a^	0.414
Gender				
Male	30 (83.3)	28 (80.0)	0.132^b^	0.717
Female	6 (16.7)	7 (20.0)		
Marital Status				
Unmarried	3 (8.3)	5 (14.3)	0.495^c^	0.871
Married	26 (72.3)	25 (71.4)		
Divorced or widowed	7 (19.4)	5 (14.3)		
Educational level				
Primary school and below	5 (13.9)	7 (20.0)	1.664^d^	0.645
Junior high school	13 (36.1)	15 (42.9)		
Senior high school	9 (25.0)	8 (22.9)		
Unior college and above	9 (25.0)	5 (14.3)		
Monthly income (RMB)				
<5,000	18 (50.0)	19 (54.3)	0.061^b^	0.806
≥5,000	18 (50.0)	16 (45.7)		
Residence				
Living alone	5 (13.9)	9 (25.7)	1.568^b^	0.211
Living with family	31 (86.1)	26 (74.3)		
Medical insurance				
Yes	30 (83.3)	28 (80.0)	0.132^b^	0.717
No	6 (16.7)	7 (20.0)		
NYHA functional classification				
I	5 (13.9)	6 (17.1)	0.638^d^	0.523
II	15 (41.7)	16 (45.7)		
III	16 (44.4)	13 (37.2)		
EF (%)	49.28 ± 6.49	50.00 ± 6.28	0.476^a^	0.635
Course of disease (years)	0.92 (0.25, 2.92)	0.92 (0.83,2.98)	1.614^e^	0.101
Depression				
Yes	7 (19.4)	11 (32.4)	1.525^b^	0.217
No	29 (80.6)	23 (67.7)		
QoL (score)	71.86 ± 6.12	72.91 ± 6.59	0.698^a^	0.635
Self-care behaviors (score)	95.44 ± 6.40	97.00 ± 6.56	1.011^a^	0.315

^a^Independent sample *t*-test; ^b^Chi-square test; ^c^Fisher’s exact test; ^d^Wilcoxon rank sum test; ^e^Mann–Whitney U test. CHF, Chronic heart failure; EF, Ejection fraction; NYHA, New York Heart Association; QoL, Quality of life.

**Table 3:** Comparison of baseline data between the two groups of caregivers.

Variables	Caregiver		Test value	*p* value
	Intervention group (*n* = 36)	Control group (*n* = 35)		
Age (years)	61.00 (53.00,66.75)	60.00 (50.00,66.00)	1.112^a^	0.266
Gender				
Male	13 (36.1)	16 (45.7)	0.677^b^	0.411
Female	23 (63.9)	19 (54.3)		
Relationship with patients				
Spouse	23 (63.9)	18 (51.4)	2.765^c^	0.248
Son or daughter	7 (19.4)	13 (37.2)		
Other relative	6 (16.7)	4 (11.4)		
Self-reported health				
Good	12 (33.3)	12 (34.3)	0.522^d^	0.617
Fair	18 (50.0)	20 (57.1)		
Bad	6 (16.7)	3 (8.6)		
Living with patients				
Yes	31 (86.1)	28 (80.0)	0.472^b^	0.492
No	5 (13.9)	7 (20.0)		
Residence				
Living alone	5 (13.9)	9 (25.7)	1.568^b^	0.211
Living with family	31 (86.1)	26 (74.3)		
Monthly income (RMB)				
<5,000	24 (66.7)	24 (68.6)	0.029^b^	0.864
≥5,000	12 (33.3)	11 (31.4)		
Educational Level				
Primary school and below	5 (13.9)	9 (20.0)	0.258^d^	0.797
Junior high school	15 (41.7)	11 (31.4)		
Senior high school	9 (25.0)	7 (20.0)		
Unior college and above	7 (19.4)	10 (28.6)		
Work				
Yes	7 (19.4)	11 (31.4)	1.347^b^	0.285
No	29 (80.6)	24 (68.6)		
Care burden (score)	67.28 ± 4.77	68.20 ± 4.38	0.848^e^	0.399

^a^Mann–Whitney U test; ^b^Chi-square test; ^c^Fisher’s exact test; ^d^Wilcoxon rank sum test; ^e^Independent sample *t*-test.

### 3.2 Comparing the QoL between the two patient groups

Two independent samples t-test was chosen for analysis. The results demonstrated significantly lower QoL scores in the intervention group compared to controls at both T1 and T2 timepoints (all *p* < 0.05). Repeated measures ANOVA was conducted to examine longitudinal changes in total QoL scores between patient groups. The results of the within-subject effect test showed that the time effect (*F*-time = 164.64), inter-group effect (*F*-intergroup = 83.33), and interaction effect (*F*-interaction = 87.46) of the QoL scores in the two groups of patients all showed statistically significant differences (*p* < 0.05). Details are shown in Table 4. The primary outcome measure of this study was QoL, which was used to evaluate the effectiveness of the intervention. We employed multivariate linear regression analysis to control for potential confounding factors and determine the impact of home-based disease management interventions on the QoL (MLHFQ scores) of elderly CHF patients at T1 and T2. A regression model is constructed using the stepwise regression method. Based on a literature review and preliminary statistical analysis of QoL in demographic and disease characteristics (Supplementary Table 1), potential confounding factors that may influence QoL were identified and incorporated into the model. The results of multiple linear regression indicated that, after controlling for baseline age, educational attainment, monthly income, gender, QoL, depression, and self-management behaviors, the intervention had a significant effect on QoL scores at T1 [*B* = -6.855, 95% *CI* (−10.200, −3.510), *p* < 0.001]. This indicates that, compared to the control group, the intervention in the experimental group reduced the patients’ average QoL score by 6.855 points. The adjusted *R*^2^ for the model was 0.414, meaning that the model explained 41.4% of the variance in QoL. In addition, the intervention had a significant effect on the QoL score at T2 [*B* = -25.00, 95% *CI* (−27.825, −22.175), *p* < 0.001]. This indicates that, compared to the control group, the intervention in the experimental group reduced the patients’ average QoL score by 25.00 points. The adjusted *R*^2^ for the model was 0.828, meaning that the model explained 82.8% of the variance in QoL. Detailed results are presented in Table 5.

**Table 4:** Comparison of total scores for QoL, self-care behavior, and care burden between the two groups.

Variables	Group	T0	T1	T2	*F_time_* value	*F_Intergroup_* value	*F*_Interaction_ value
QoL	Intervention group (*n* = 36)	71.86 ± 6.12	53.56 ± 8.62^a^	44.14 ± 5.12^a,b^	164.64	83.33	87.46
	Control group (*n* = 35)	72.91 ± 6.59	61.26 ± 8.11^a^	69.89 ± 7.34^b^	–	–	–
	*t* value	0.698	3.873	17.107	–	–	–
	*p* value	0.487	<0.001	<0.001	<0.001	<0.001	<0.001
Self-care behavior^#^	Intervention group (*n* = 36)	95.44 ± 6.40	116.92 ± 1.67^a^	107.92 ± 1.42 ^a,b^	89.03	16.95	34.33
	Control group (*n* = 35)	97.00 ± 6.56	102.03 ± 1.69^a^	99.63 ± 1.44	–	–	–
	*t* value	1.011	6.304	4.106	–	–	–
	*p* value	0.315	<0.001	<0.001	<0.001	<0.001	<0.001
Care burden	Intervention group (*n* = 36)	67.28 ± 4.77	36.89 ± 9.06^a^	43.08 ± 7.44^a,b^	311.02	40.63	10.41
	Control group (*n* = 35)	68.20 ± 4.38	48.80 ± 10.35^a^	49.86 ± 9.42^a^	–	–	–
	*t* value	0.848	5.165	3.367	–	–	–
	*p* value	0.399	<0.001	0.001	<0.001	<0.001	<0.001

T0: pre-intervention (baseline); T1: at the end of the intervention; T2: 3 months after the end of the intervention. ^a^Compared with T0, *p* < 0.05*. ^b^Compared with T1, *p* < 0.05*. ^#^Self-care behavior is measured using the SCHFI tool.

**Table 5:** Multiple linear regression analysis of QoL at T1 and T2 for home-based disease management interventions.

Variables	QoL (T2)			QoL (T3)		
	*B*	*p* value	95% confidence interval	*B*	*p* value	95% confidence interval
Received home-based disease management intervention (Yes)	−6.855	**<**0.001	−10.200, −3.510	−25.00	**<**0.001	−27.825, −22.175
QoL(T0)	0.513	**<**0.001	0.247, 0.779	0.324	0.005	0.099, 0.549
Monthly income (≥ 5,000 Yuan)	−7.138	**<**0.001	−10.479, −3.796	−3.452	0.017	−6.274, −0.630

T0: pre-intervention (baseline); T1: at the end of the intervention; T2: 3 months after the end of the intervention.

### 3.3 Comparing the readmission rates of the two patient groups

The chi-square test was used to statistically assess the readmissions, and the results revealed that at the finish of the intervention (T1), the intervention group’s readmission rate was significantly lower than the control groups, and the difference was statistically significant (*p* = 0.027). At 3 months after the end of the intervention (T2), the readmission rate of the intervention group was also significantly lower than that of the control group, and the difference was statistically significant (*p* = 0.033). See Table 6 for details.

**Table 6:** Comparison of readmission and depression between the two groups.

Time	Items	Intervention group (*n* = 36)	Control group (*n* = 35)	*c*^2^ value	*p* value
T1	Readmission (Yes)	3(8.3)	10(28.6)	4.860	0.027
	Depression (Yes)	3(8.3)	10(28.6)	4.860	0.027
T2	Readmission (Yes)	7(19.4)	15(42.9)	4.549	0.033
	Depression (Yes)	6(16.7)	15(42.9)	5.844	0.016

T1: at the end of the intervention; T2: 3 months after the end of the intervention.

### 3.4 Comparing the self-management behaviors of the two patient groups

The t-test for two independent samples was employed for analysis. The findings showed that patients in the intervention group had higher overall self-care behavior scores at T1 and T2 than patients in the control group at each time point, and the difference was statistically significant (*p* < 0.05). Repeated measures ANOVA was used to compare the differences in the total scores of self-care behaviors between the two groups of patients at different time points. The multivariate test results indicated that the time effect (*F*-time = 89.03), between-group effect (*F*-intergroup = 16.95), and interaction effect (*F*-interaction = 34.33) of the total self-care behavior scores in both groups showed statistically significant differences (*p* < 0.05). Details are presented in Table 4.

### 3.5 Comparing the depression of two patient groups

The results displayed that after the intervention, at T1, the incidence of depression in the intervention group was significantly lower than that in the control group (*p* = 0.027). At T2, the intervention group exhibited significantly lower incidence rates of depression compared to the control group (*p* = 0.016). Details are presented in Table 6.

### 3.6 Comparing the care burden of the two caregivers

Two independent samples t-test was used for analysis. The outcomes demonstrated that at T1 and T2, the total care burden scores of caregivers in the intervention group were lower than those of the control group at each time point, and the difference was statistically significant (*p* < 0.05). A repeated measures ANOVA was conducted to compare between-group differences in total caregiver burden scores across time points. The results of the multivariate test showed that the time effect (*F*-time = 311.02), the between-group effect (*F*- Intergroup = 40.63), and the interaction effect (*F*-interaction = 10.41) of the two care burden scores showed a statistically significant difference (*p* < 0.05), and the details are given in Table 4.

## 4 Discussion

The significantly lower Minnesota Living with Heart Failure Questionnaire (MLHFQ) total scores in the intervention group at both T1 and T2, coupled with a progressive improvement in their QoL, underscores the sustained benefit of the home-based, dyadically-focused management program. In addition, regression models confirmed the strong, sustained effect of the home-based intervention on QoL, which increased over time, indicating a cumulative benefit. This intervention effect persisted even as the protective influence of higher income diminished, suggesting the program may mitigate socioeconomic disparities. These results are consistent with the findings by Xiao et al. (37) in their breast cancer patient study. Foreign scholars have similarly suggested in the study that disease-specific education and guidance on dealing with symptoms are important for patients and their informal caregivers to cope with the disease and improve their QoL (38).

The efficacy of our intervention can be explained by its core mechanism: it systematically targets the dyadic relationship as the engine for sustainable health outcomes. From hospitalization through the post-discharge phase, the intervention reinforced a collaborative model where the functions of the patient and caregiver were complementary. By implementing synchronized education and rehabilitation guidance, we empowered both parties-enhancing the caregiver’s professional skills and illness coping abilities while providing the patient with more comprehensive support. This reciprocal process likely breaks the cycle of mutual distress and fosters a positive feedback loop of shared efficacy. This approach aligns with the core tenets of ideal home care as outlined by the Agency for Healthcare Research and Quality (39), which emphasizes meeting the needs of the care unit. Our study demonstrates that a paradigm shift from patient-centric to dyad-centric care is not only feasible but necessary for optimizing long-term outcomes in elderly CHF management. By addressing the patient-caregiver dyad as an integrated unit, healthcare providers can leverage this natural partnership to achieve a more profound and durable impact than by focusing on the patient alone.

The significantly lower readmission rate observed in the intervention group at both T1 and T2 can be attributed to the comprehensive, dyadically-structured intervention that transcended conventional patient education. While consistent with prior research on home-based management for chronic conditions (40) and systematic reviews affirming the value of intensive education (41, 42), our study elucidates a more nuanced mechanism: the reduction in readmission was likely mediated by the establishment of a coherent, collaborative patient-caregiver system. The efficacy of our intervention stems from its dual-focused strategy, which simultaneously augmented the capabilities of both patients and their informal caregivers. For patients, this involved enhancing self-management cognition, symptom recognition, and practical skills, fostering a more proactive understanding of their disease trajectory. For caregivers, systematic training clarified their roles and enhanced their supervisory and guidance competencies. Critically, by aligning the cognition and behaviors of both parties through a shared framework, the intervention mitigated dyadic incongruence-a state where discrepancies in illness perceptions and care goals between patients and caregivers lead to conflict and ineffective management (43). This reduction in dyadic stress and improvement in collaborative problem-solving likely served as the key mechanism preventing clinical deteriorations that typically precipitate readmission. This collaborative dyadic model breaks the cycle of readmissions by empowering the patient-caregiver unit, offering a cost-effective strategy for improving post-discharge outcomes in elderly CHF patients.

The sustained superiority of self-care behaviors in the intervention group at both T1 and T2, despite a post-intervention decline from peak levels, indicates that the dyadic intervention fostered a fundamental internalization of management skills rather than inducing only short-term compliance. This pattern of a high initial gain followed by stabilization at a level significantly above baseline aligns with Lu′s findings on the importance of skill consolidation (44), and contrasts with studies reporting a steeper decline after intervention cessation (43). In the process of home-based disease management for elderly patients with CHF, patients and informal caregivers often have cognitive differences in dimensions such as symptom recognition, cognition of self-care, medical decision-making, and confidence in self-care. This inconsistency will lead to insufficient participation of both parties in disease management, which in turn will cause the problem of reduced adherence to self-care in patients. By treating the dyad as an integrated unit and combining HF education with congruence assessment and collaborative training, our program facilitated a shared illness representation and unified action plan. This alignment likely transformed the dyad from a potential source of conflict into a cohesive, mutually reinforcing team, which is essential for maintaining complex self-care regimens over time. Furthermore, the program’s extended, follow-up provided a critical scaffold for this process. The longitudinal support allowed for the refinement and application of learned skills in the home environment, enabling dyads to proactively identify symptoms and initiate management strategies effectively, thereby preventing clinical deterioration (16). This continuous reinforcement mechanism appears to have been pivotal in translating initial behavioral gains into durable self-management capacity, suggesting that the sustainability of such interventions is contingent not only on their content but also on the duration and quality of post-instructional support.

Depression in heart failure patients are frequently underrecognized, yet they significantly impede clinical recovery and contribute to adverse long-term outcomes (45). The significantly lower incidence of depression in the intervention group at both T1 and T2 underscores the psychological benefits of a dyadic management approach. This finding aligns with prior research by Li (46) and extends it by revealing the potential mechanisms through which a structured, home-based program can mitigate depressive symptoms in elderly CHF patients. We propose that this psychological benefit was mediated by a dual-pathway mechanism. First, the intervention enhanced patients’ self-perceived health status-a known predictor of depression trajectory (47), by systematically equipping them and their caregivers with practical disease management skills. The ability to accurately recognize symptom changes and implement timely, correct coping measures fostered a sense of control and self-efficacy, directly countering feelings of helplessness and anxiety about disease progression. Second, the program strengthened the caregiver’s capacity to provide competent, scientifically grounded support. This not only improved the practical management of the illness but also significantly enhanced the patient’s psychological security by creating a reliable and effective support system (48).

The results of this study revealed that at both T1 and T2 assessment time points, the Zarit Burden Interview (ZBI) scores of informal caregivers in the intervention group were lower than those in the control group (*p* < 0.05). This is in line with a study conducted abroad (49). The daily unpredictability of heart failure (i.e., acute exacerbations and hospitalizations), medication burden and side effects, and patient overdependence result in a non-negligible caregiving burden for caregivers, which may contribute to caregivers becoming somewhat less engaged in the management of the patient’s disease or even abandoning the caregiving role (50). Our intervention directly counteracted these challenges by maintaining substantial, high-quality communication with caregivers. Through continuous education, value clarification, and responsive support, we facilitated their successful transition into a “skilled caregiver” role. This empowerment enabled them to perform caregiving tasks with greater competence and confidence, thereby reducing the physical and psychological strain associated with unmanaged complexities. In addition, this study, which focused on CHF patients and their caregivers as the intervention unit, significantly improved the concordance of both parties’ assessment of clinical symptoms, including shortness of breath, fatigue, pain, and edema. When patients and caregivers converged on symptom perceptions, their caregiving collaboration was more coordinated, which not only improved caregiving experience and outcomes, but also reduced the risk of negative emotions for both partners. This synergistic effect prompted patients and caregivers to engage in home heart failure management together with a more positive and optimistic attitude, thus effectively alleviating the caregiver’s burden of care.

## 5 Conclusion

This study developed a home-based disease management program that demonstrates multiple clinical benefits for elderly patients with CHF: it significantly improves QoL, reduces readmission rates, enhances self-management capacity, and effectively alleviates negative emotions. Notably, the program also shows remarkable efficacy in reducing caregiving burden for informal caregivers. These findings provide new practical evidence and intervention strategies for long-term management of CHF and optimization of home-based care models for chronic diseases. Disease management for CHF patients is a long-term process, during which both patients and caregivers may encounter new challenges at different stages. Continuous follow-up and timely support are crucial for sustaining intervention effects. However, due to limitations in time and human resources, this study only assessed outcomes at 3 months post-intervention. Additionally, participants were recruited from only one tertiary hospital in Jiangsu Province, which may not fully account for cultural differences and varying levels of acceptance. In future studies, we will enlarge the sample size, extend the intervention and follow-up duration, and actively explore dyadic intervention models tailored to China’s specific healthcare context, with the aim of further refining and validating our findings in subsequent research.

## 6 Limitation

However, due to limitations in time and human resources, this study only assessed outcomes at 3 months post-intervention. Additionally, participants were recruited from only one tertiary hospital in Jiangsu Province, which may not fully account for cultural differences and varying levels of acceptance. In future studies, we will enlarge the sample size, extend the intervention and follow-up duration, and actively explore dyadic intervention models tailored to China’s specific healthcare context, with the aim of further refining and validating our findings in subsequent research. Currently, high-quality research on dual interventions is scarce. This restricts the evidence base for translating dual disease management interventions targeting elderly patients with chronic heart failure who live at home and their informal caregivers into widespread practice. Further research is needed into the mechanisms through which dual interventions influence disease management behaviors among patients and informal caregivers.

## Data Availability

The raw data supporting the conclusions of this article will be made available by the authors, without undue reservation.
