# Supplementary material for: Effects of home disease management strategies based on the dyadic illness management theory on elderly patients with chronic heart failure and informal caregivers’ physical and psychological outcomes: a randomized controlled trial
Source: Front Med (Lausanne). 2025 Nov 18;12:1679743. doi: 10.3389/fmed.2025.1679743 (PMC12669140; doi:10.3389/fmed.2025.1679743)
Supplement: Supplementary file 1 [file Table_1.DOCX]

**Analysis of the quality of life at the T1 and T2 time points in terms of demographic characteristics and disease characteristics**

| **Variables** | **QoL (T1)** | ***P Value*** | **QoL (T2)** | ***P Value*** |
| --- | --- | --- | --- | --- |
| **Age (years)** | 0.159 | 0.186 | 0.168 | 0.162 |
| **Body mass index (kg/m^2^)** | -0.116 | 0.334 | -0.115 | 0.341 |
| **Ejection fraction(%)** | -0.078 | 0.520 | -0.039 | 0.748 |
| **Course of disease (years)** | -0.156 | 0.194 | -0.145 | 0.229 |
| **Self-care behavior (scores)** | -0.403 | ＜0.001 | -0.405 | ＜0.001 |
| **Received home-based disease management intervention (Yes)** | 53.56±8.62 | ＜0.001 | 44.14±5.12 | ＜0.001 |
| **Gender** |  |  |  |  |
| Male | 58.02±9.51 | 0.199 | 56.74±14.39 | 0.964 |
| Female | 54.38±7.04 |  | 56.54±14.61 |  |
| **Monthly income (RMB)** |  |  |  |  |
| <5000 | 60.73±9.15 | ＜0.001 | 58.78±13.93 | 0.210 |
| ≥5000 | 55.68±7.78 |  | 54.47±14.62 |  |
| **Residence** |  |  |  |  |
| Living alone | 57.86±9.05 | 0.820 | 60.14±15.35 | 0.320 |
| Living with family | 57.23±9.28 |  | 55.86±14.08 |  |
| **Medical insurance** |  |  |  |  |
| Yes | 58.02±9.51 | 0.199 | 56.74±14.39 | 0.964 |
| No | 54.38±7.042 |  | 56.54±14.609 |  |
| **Readmission** |  |  |  |  |
| No | 56.03±7.85 | 0.010 | 53.65±14.14 | 0.006 |
| Yes | 63.23±12.34 |  | 63.52±12.51 |  |
| **Depression** |  |  |  |  |
| No | 56.17±7.85 | 0.101 | 53.40±14.09 | 0.002 |
| Yes | 62.62±12.73 |  | 64.57±11.805 |  |
| **Marital Status** |  |  |  |  |
| Unmarried | 62.25±7.07 | 0.165 | 58.38±15.00 | 0.912 |
| Married | 57.12±9.00 |  | 56.96±13.90 |  |
| Divorced or widowed | 55.08±10.32 |  | 54.50±16.62 |  |
| **Educational Level** |  |  |  |  |
| Primary school and below | 54.83±5.49 | 0.624 | 57.67±13.91 | 0.899 |
| Junior high school | 56.89±9.00 |  | 57.11±15.65 |  |
| Senior high school | 58.24±10.53 |  | 57.53±15.49 |  |
| Unior college and above | 59.36±10.55 |  | 54.07±11.29 |  |
| **NYHA functional classification** |  |  |  |  |
| Ⅰ | 57.73±11.97 | 0.929 | 53.91±14.46 | 0.781 |
| Ⅱ | 56.87±8.20 |  | 57.03±13.96 |  |
| Ⅲ | 57.72±9.32 |  | 57.41±15.02 |  |
